# Investigating the relationship between self-perception of fracture risk and prior fracture: findings from the Hertfordshire Cohort Study

**DOI:** 10.1007/s40520-022-02322-6

**Published:** 2022-12-19

**Authors:** Gregorio Bevilacqua, Leo D. Westbury, Ilse Bloom, Jean Zhang, Kate A. Ward, Cyrus Cooper, Elaine M. Dennison

**Affiliations:** 1 Medical Research Council (MRC) Lifecourse Epidemiology Centre, University of Southampton, Southampton General Hospital, Southampton, UK; 2 National Institute for Health and Care Research (NIHR), Southampton Biomedical Research Centre, University of Southampton and University Hospital Southampton National Health Service (NHS) Foundation Trust, Southampton, UK; 3 National Institute for Health and Care Research (NIHR), Oxford Biomedical Research Centre, University of Oxford, Oxford, UK; 4 School of Biological Sciences, Victoria University of Wellington, Wellington, New Zealand

**Keywords:** Self-perceived risk of fracture, Fracture, Social isolation, Self-efficacy, Mental health, Older adults

## Abstract

**Background:**

Self-perceived risk of fracture (SPR) is associated with fracture independent of FRAX calculated risk. To understand this better we considered whether lifestyle factors not included in the FRAX algorithm and psychosocial factors (social isolation, self-efficacy, or mental health status) explain the relationship between SPR and fracture.

**Methods:**

We studied 146 UK community-dwelling older adults from the Hertfordshire Cohort Study. SPR ranked as ‘lower’, ‘similar’ and ‘higher’ relative to others of the same age, was assessed by questionnaire. Social isolation was assessed using the six-item Lubben Social Network Scale; self-efficacy was assessed using a shortened General Self-Efficacy Scale (GSE); mental health status was assessed using the anxiety/depression item from the EuroQoL questionnaire. SPR in relation to previous self-reported fracture was examined using logistic regression.

**Results:**

Among participants of median age 83.4 (IQR 81.5–85.5) years, SPR was lower for 54.1% of participants, similar for 30.8%, and higher for 15.1%; 74.7% reported no previous fractures. Greater SPR was associated with increased odds of previous fractures when adjusting for sex and age only (OR 1.72, 95% CI 1.03–2.87, per higher band of SPR). While further individual adjustment for social isolation (1.73, 1.04–2.89), self-efficacy (1.71, 1.02–2.85), or mental health (1.77, 1.06–2.97) did not attenuate the relationship, individual adjustment for diet quality and number of comorbidities did.

**Conclusions:**

Adjustment for social isolation, self-efficacy or mental health status did not attenuate the relationship between SPR and fracture. By contrast, lifestyle factors not included in FRAX, such as diet quality, did attenuate relationships, suggesting a possible future area of investigation.

## Introduction

Almost one in two women and one in four men after the age of 50 years are at risk of any osteoporotic fracture [1, 2]. As life-expectancy increases, resulting in increases in the ageing population, health and economic burdens are likely to escalate. The fracture risk assessment tool FRAX is widely used to calculate individuals’ 10-year probability of incurring hip or other major osteoporotic fractures [3]. The FRAX tool takes into account a number of known risk factors such as age, sex, body mass index (BMI), history of fractures, smoking habits, use of glucocorticoids, rheumatoid arthritis, and secondary osteoporosis; bone mineral density (BMD) is an optional variable in the algorithm [4, 5].

Self-perceived risk of fracture (SPR) is a subjective perception of one’s risk of experiencing a fracture compared to others of the same age and sex. It has been previously suggested that SPR may capture aspects important for fracture risk but which are not accounted for in current prediction tools such as FRAX [2, 6]. In previous work with women aged 55 years and older, we and others have found that higher SPR was associated with improved uptake of anti-osteoporosis medications and poorer skeletal parameters (e.g., lower radial trabecular volumetric density and number, higher trabecular separation, and lower tibial cortical area), and importantly that both the FRAX-calculated level of risk and SPR had a significant, independent association with fracture [2, 6]

Socio-psychological factors are recognised as important to health, and specifically musculoskeletal health. Previous studies with older adults have found social isolation (i.e., the scarceness or absence of regular social contacts and relationships with others), poor self-efficacy (i.e., one’s poor confidence in the ability to cope with the demands, tasks and challenges of life), depression, and multimorbidity to be associated with poor self-rated health [7–10]. Social isolation has been found to increase the risk of becoming physically frail in English community-dwelling men aged 60 years and older [11] and to be associated with a higher incidence of hip fractures among Israeli patients aged 65 years and older who lived alone during the COVID-19 lockdown [12]. A study conducted among US community-dwelling adults, aged approximately 72 years, identified self-efficacy as a predictor of falls risk, with higher self-efficacy being associated with the adoption of health behaviours (diet and physical activity) known to be preventive of falls [13]. Finally, several studies reported associations between depression and increased risk of fracture in different populations [14–17].

For these reasons, in this study we considered whether these same factors (social isolation, general self-efficacy, and mental health status) were associated with SPR among participants from a cohort of English community-dwelling older adults and examined whether they explained the relationship between SPR and risk of prior fracture. In addition, a number of lifestyle factors are recognised as important in the pathogenesis of low bone density but are not currently included in FRAX. These include diet [18–24], physical activity [25, 26], and medical history (where the clinician does not consider the patient to have secondary osteoporosis) [27, 28]. For this reason, we also examined relationships between these factors and SPR and investigated whether they explained the relationship between SPR and previous fracture. Finally, we also considered relationships between current bisphosphonate use and SPR, as these have been reported in previous studies [6].

## Methods

Participants were recruited from the Hertfordshire Cohort Study (HCS), a population-based sample of men and women born between 1931 and 1939 in Hertfordshire, UK, and who still lived there in 1998–2004 when they completed a nurse-administered home visit and clinic visit for a detailed characterisation of their health. These participants were originally recruited to study the relationship between growth in infancy and the subsequent risk of adult diseases [29, 30].

Between November 2019 and March 2020, 176 participants from the HCS (94 men and 82 women) took part in a follow-up study. They were visited at home by a trained fieldworker who administered a questionnaire that included information on medical history, medication use, lifestyle and social isolation. The visits also included measurements of height and weight to calculate body mass index (BMI).

SPR was assessed by asking participants to rate their risk of fracturing/breaking a bone, compared to other men and women of the same age; responses (‘much lower’; ‘a little lower’; ‘about the same’; ‘a little higher’; and ‘much higher’) were categorised as: ‘lower’, ‘similar’ and ‘higher’. Fracture history since age 45 years was ascertained from a previous 2017 follow-up of the HCS via a fieldworker-administered questionnaire of fractures since age 45; in addition, in 2019–2020, participants were asked to report any fracture they experienced in the previous 12 months. Self-reported fractures were thus ascertained by combining answers from both questionnaires.

Social isolation was assessed using the 6-item Lubben Social Network Scale (LSNS-6) which has been validated to assess social networks and social support and to screen for social isolation in older people [31]. The LSNS-6 tool assesses the number and frequency of social interactions with friends (three items) and family members (three items). Each answer is assigned a score ranging from 0 (“none”) to five (“nine or more”), and the overall final score ranges from 0 (indicating high isolation or few social resources) to 30 (indicating low isolation or many social resources). Participants were identified as socially isolated if they had an LSNS-6 score of < 12, in accordance with Lubben and colleagues [31]. The LSNS-6 has been shown to have good internal consistency across samples of community-dwelling older adults [31–33].

General self-efficacy was assessed using a shortened version of the Generalised Self-Efficacy Scale (GSE) developed by Schwarzer and Jerusalem in 1981 to measure optimistic self-belief in coping with the demands, tasks and challenges of life in general; the scale has been proved to have good psychometric properties [34]. While the original GSE consists of 10 items, in this study we used a 5-item version which, in a large Norwegian study, has shown to have high internal consistency (Cronbach’s α of 0.83) [35]. The shortened GSE consists of the following items: “I can always manage to solve difficult problems if I try hard enough”; “I can find a way to get what I want even if someone is trying to stop me”; “It is easy for me to stick to my aims and reach my goals”; “I am calm when things are difficult because I know I can cope”; and “If I am in trouble I can usually find a way out”. Each item has four possible answers ranging from ‘strongly disagree’ (to which the lowest value of 1 is assigned, indicating low efficacy) to ‘strongly agree’ (to which the highest value of 4 is assigned, indicating high efficacy). For the current study, we treated the GSE score as an untransformed continuous variable.

Mental health was assessed using the anxiety/depression dimension of the EuroQol 5-Dimension (EQ-5D) questionnaire, a widely used and validated instrument designed to measure health-related quality of life [36, 37]. For the EQ-5D anxiety/depression dimension, participants are asked to indicate what of the following statements best describes their quality of life: (i) “I am not anxious or depressed”; (ii) “I am moderately anxious or depressed”; and (iii) “I am extremely anxious or depressed”. For the current study, we combined (ii) and (iii) and generated a binary variable (“not anxious/depressed” vs “anxious/depressed”).

The questionnaire also recorded a number of lifestyle factors. Smoker status was categorised as ‘never smoked’ and ‘current/ex-smoker’ depending on the participants’ answers to the questions ‘Do you currently smoke?’ and ‘Have you ever been a smoker?’. Participants were asked how often they currently drank different types of alcohol (beer, wine, spirits, etc.) and how much they normally drank each time. This was used to estimate their alcohol consumption in units per week. Physical activity time was assessed using the Longitudinal Aging Study Amsterdam (LASA) physical activity questionnaire (LAPAQ) and calculated as the average amount of time (in minutes per day) spent walking outside, cycling, gardening, playing sports or doing housework in the last 2 weeks. The LAPAQ was shown to be highly correlated with a seven-day diary (*r* = 0.68; *p* < 0.001) and moderately correlated with a pedometer (*r* = 0.56; *p* < 0.001) [38]. A food frequency questionnaire (FFQ) was used to calculate a ‘prudent’ diet score, based on participants’ consumption of 24 indicator foods; the score was used as a measure of diet quality. Higher prudent diet scores indicate healthier diets, characterised by higher consumption of fruit, vegetables, whole grain cereals, and oily fish and lower consumption of white bread, added sugar, full-fat dairy products, chips, and processed meat [39, 40].

We also assessed the number of comorbidities by asking the question: ‘Have you been told by a doctor that you have any of the following conditions?’. The following conditions were recorded: high blood pressure, diabetes, lung disease (asthma, COPD, emphysema, chronic bronchitis), rheumatoid arthritis, multiple sclerosis, cancer, vitiligo, depression, Parkinson’s disease, heart disease (heart attack, angina, heart failure), peripheral arterial disease (claudication), osteoporosis, thyroid disease, and stroke.

Finally, we assessed the use of bisphosphonates (drugs used to prevent or reduce the risk of osteoporosis) by asking participants to indicate whether they were taking any regular medications.

### Statistical methods

Participant characteristics were described using means and standard deviations (SD), medians and inter-quartile ranges (IQR) and frequency and percentage distributions among the whole sample and stratified by SPR. Associations between SPR and previous fracture were examined using logistic regression models with the following sets of adjustments: sex and age; additionally adjusted for each of the psychosocial factors in turn; additionally adjusted for BMI, smoking status, alcohol consumption, physical activity, prudent diet score, number of comorbidities, and current bisphosphonate use. These adjustments were included as the use of osteoporosis medications was related to SPR in previous studies [2, 6], and the other non-psychosocial adjustments are widely known to influence fracture risk or bone health in general [41, 42], so could act as potential confounders in the relationship between SPR and previous fracture. To determine whether any of these psychosocial or lifestyle factors explain the relationship between SPR and previous fracture, each individual factor was examined in relation to previous fracture and SPR using logistic and ordinal regression, respectively, with sex and age included as adjustments in all models, with a particular focus on the factors not included in the FRAX algorithm.

To examine whether the relationship between SPR and previous fracture differed according to different values of the psychosocial characteristics (LSNS-6 score, GSE score and EuroQol anxiety/depression), separate logistic regression models with previous fracture as the outcome were fitted for each of these psychosocial characteristics which included an interaction term between SPR and the psychosocial characteristic as well as their main effects, along with sex and age.

To maintain sample size, men and women were pooled (sex-interaction effects were not statistically significant) and all analyses were adjusted for sex; *p* < 0.05 was regarded as statistically significant. Analyses were conducted using Stata, release 17.0. The analysis sample comprised 146 participants with data on previous fracture, SPR and at least one of the following: LSNS-6 score, GSE score and EuroQol anxiety/depression.

## Results

### Participant characteristics

Table 1 provides the characteristics of the participants. The median (IQR) age of the analysis sample was 83.4 (81.5, 85.5) years. The number of participants who described their SPR as ‘lower’ compared to other individuals of the same age and sex was 79 (54.1%); 45 (30.8%) and 22 (15.1%) described their SPR as ‘similar’ and ‘higher’, respectively. Overall, 25.3% reported a previous fracture; this increased from 17.7% in the lowest SPR category to 36.4% in the highest. In the analysis sample, 26 (17.8%) were socially isolated, 35 (24.1%) were anxious or depressed, and the mean (SD) GSE score was 14.9 (1.9).

**Table 1:** Participant characteristics of the analysis sample as a whole and stratified by self-perceived risk of fracture

Participant characteristic	All (*n* = 146)	Self-perceived risk of fracture		
		Lower (*n* = 79)	Similar (*n* = 45)	Higher (*n* = 22)
Age (years)*	83.4 (81.5, 85.5)	83.4 (81.4, 85.4)	83.4 (81.7, 86.0)	82.8 (81.2, 85.3)
Female sex‡	70 (47.9%)	36 (45.6%)	23 (51.1%)	11 (50.0%)
Height (cm)†	164.7 (9.1)	166.0 (9.4)	163.0 (8.4)	163.7 (9.5)
Weight (kg)†	74.1 (13.5)	75.0 (14.3)	72.2 (12.1)	74.6 (13.7)
BMI (kg/m^2^)†	27.2 (4.1)	27.0 (3.9)	27.2 (4.1)	27.7 (4.7)
Ever smoked regularly‡	58 (40.0%)	26 (33.3%)	22 (48.9%)	10 (45.5%)
Alcohol consumption (units per week)*	1.8 (0.0, 7.0)	1.5 (0.0, 7.0)	2.3 (0.0, 8.1)	1.5 (0.0, 8.3)
Activity time in last 2 weeks (min/day) [LAPAQ]*	128.2 (77.1, 182.1)	135.4 (84.3, 188.6)	116.8 (66.8, 175.7)	126.4 (57.9, 171.4)
Prudent diet score†	0.0 (1.4)	0.3 (1.4)	− 0.3 (1.2)	− 0.4 (1.3)
Taking bisphosphonates‡	15 (10.3%)	4 (5.1%)	7 (15.6%)	4 (18.2%)
Number of comorbidities*	2.0 (1.0, 3.0)	1.0 (1.0, 2.0)	2.0 (1.0, 3.0)	2.0 (2.0, 3.0)
SPR compared to others‡				
Lower	79 (54.1%)	79 (100.0%)	0 (0.0%)	0 (0.0%)
Similar	45 (30.8%)	0 (0.0%)	45 (100.0%)	0 (0.0%)
Higher	22 (15.1%)	0 (0.0%)	0 (0.0%)	22 (100.0%)
Self-reported fracture‡^1^	37 (25.3%)	14 (17.7%)	15 (33.3%)	8 (36.4%)
LSNS-6 score*	17.5 (14.0, 22.0)	17.0 (14.0, 22.0)	19.0 (14.0, 22.0)	17.5 (13.0, 21.0)
LSNS-6 score < 12‡	26 (17.8%)	13 (16.5%)	8 (17.8%)	5 (22.7%)
GSE score†	14.9 (1.9)	15.0 (2.1)	14.9 (1.7)	14.5 (1.9)
EuroQoL anxiety or depression‡	35 (24.1%)	21 (26.9%)	11 (24.4%)	3 (13.6%)

*Median (IQR), †Mean (SD), ‡*N* (%)

^1^Obtained from fractures since 45 years of age (ascertained in 2017) and fractures in the previous 12 months (ascertained in 2019–2020); all other characteristics were ascertained in 2019–2020

*LAPAQ* Longitudinal Aging Study Amsterdam Physical Activity Questionnaire, *LSNS-6* 6-item Lubben Social Network Scale, *GSE* Generalised Self-Efficacy Scale

### Associations between self-perception of fracture risk and previous fracture

Cross-sectional associations between SPR and previous fracture after adjustments are presented in Table 2. As expected, higher levels of SPR were associated with increased odds of having previously experienced a fracture (OR 1.72, 95% CI 1.03–2.87, *p* = 0.037, per higher band of SPR) when adjusting for sex and age only. The association remained robust to further individual adjustment for social isolation status (1.73, 1.04–2.89, *p* = 0.035), GSE score (1.71, 1.02–2.85, *p* = 0.040), and EuroQol anxiety/depression status (1.77, 1.06–2.97, *p* = 0.030). However, when each of these three models were additionally adjusted for lifestyle factors (BMI, smoking, alcohol consumption, physical activity, prudent diet score), number of comorbidities and use of bisphosphonates, the association between SPR and previous fracture was attenuated (*p* > 0.1).

**Table 2:** Odds ratios (95% CI) for previous fracture per higher band of self-reported fracture risk (lower, similar, higher) after adjustment as shown

Adjustments	Adjusted models		Fully-adjusted models^1^	
	Odds ratio (95% CI)	*P* value	Odds ratio (95% CI)	*P* value
Sex and age	1.72 (1.03,2.87)	0.037	1.67 (0.88,3.17)	0.114
Sex, age and LSNS-6 score	1.73 (1.04,2.89)	0.035	1.68 (0.89,3.19)	0.111
Sex, age and GSE score	1.71 (1.02,2.85)	0.040	1.67 (0.88,3.17)	0.114
Sex, age and EuroQol anxiety/depression	1.77 (1.06,2.97)	0.030	1.70 (0.89,3.23)	0.106

^1^Fully-adjusted models additionally accounted for BMI, smoking status (ever vs never), alcohol consumption, physical activity, prudent diet score, number of comorbidities and use of bisphosphonates

LSNS-6: 6-item Lubben Social Network Scale categorised as < 12 or ≥ 12

*GSE* Generalised Self-Efficacy Scale

Table 3 presents the odds ratios for previous fracture per higher band of SPR after adjustment for individual lifestyle factors, number of comorbidities, and use of bisphosphonates. After sex, age and SPR were included in the model, the association between SPR and previous fracture was attenuated by individual adjustment for smoking status (OR 1.67, 95% CI 1.00–2.80, *p* = 0.051 per higher band of SPR, with a percentage decline of 2.9% in the OR compared to the model only adjusted for sex and age), prudent diet score (OR 1.60, 0.92–2.80, *p* = 0.099, with a percentage decline of 7.0% in OR), number of comorbidities (OR 1.65, 0.98–2.79, *p* = 0.060, with a percentage decline of 4.1% in OR), and current use of bisphosphonates (OR 1.64, 0.97–2.76, *p* = 0.063, with a percentage decline of 4.7% in OR).

**Table 3:** Odds ratios for previous fracture per higher band of self-reported fracture risk (lower, similar, higher) with adjustment for lifestyle and demographic factors as shown

Adjustments	Odds ratio (95% CI)	*P* value	Percentage decline in odds ratio compared to model with age and sex as adjustments
Age, sex	1.72 (1.03, 2.87)	0.037	
Age, sex, BMI	1.86 (1.10, 3.16)	0.021	
Age, sex, smoking status	1.67 (1.00, 2.80)	0.051	2.9%
Age, sex, alcohol consumption	1.72 (1.04, 2.87)	0.036	0.0%
Age, sex, physical activity	1.86 (1.10, 3.16)	0.021	
Age, sex, prudent diet score	1.60 (0.92, 2.80)	0.099	7.0%
Age, sex, number of comorbidities	1.65 (0.98, 2.79)	0.060	4.1%
Age, sex, current bisphosphonate use	1.64 (0.97, 2.76)	0.063	4.7%

In logistic regression models with previous fracture as the outcome, interaction effects between SPR and each of the psychosocial characteristics (LSNS-6 score, GSE score and EuroQol anxiety/depression) were not statistically significant (*p* > 0.07 for all interaction effects). This suggests that the relationship between SPR and previous fracture was not greatly modified by these psychosocial characteristics.

### Participant characteristics in relation to previous fracture and SPR

Cross-sectional associations between participant characteristics and SPR are presented in Table 4. Higher prudent diet scores were associated with decreased odds of being in a higher SPR category (OR 0.70, 95% CI 0.54–0.91, *p* = 0.007 per unit increase in prudent diet score), while higher numbers of comorbidities and current use of bisphosphonates were associated with increased odds of being in a higher SPR category (OR 1.38, 1.07–1.78, *p* = 0.013 per additional comorbidity, and OR 2.97, 1.12–7.93, *p* = 0.029 for bisphosphonate use versus no use). The marked association between diet quality and SPR was robust to adjustment for age, sex, BMI, smoking status, alcohol consumption, physical activity, number of comorbidities and current use of bisphosphonates.

**Table 4:** Odds ratios for being in a higher category of self-reported fracture risk (lower, similar, higher) according to individual participant characteristics after adjustment for sex and age

Characteristic	Odds ratio (95% CI)^1^	*P* value
LSNS-6 score (< 12 vs. ≥ 12)	1.25 (0.55, 2.85)	0.587
GSE score	0.92 (0.78, 1.10)	0.362
EuroQol anxiety/depression	0.64 (0.30, 1.37)	0.252
BMI (kg/m^2^)	1.02 (0.95, 1.11)	0.554
Smoking (ever vs never)	1.81 (0.94, 3.49)	0.074
Alcohol (units per week)	1.01 (0.98, 1.04)	0.376
Physical activity (mins/day)	1.00 (0.99, 1.00)	0.426
Prudent diet score	0.70 (0.54, 0.91)	0.007
Number of comorbidities	1.38 (1.07, 1.78)	0.013
Current use of bisphosphonates	2.97 (1.12, 7.93)	0.029

^1^Odds ratios correspond to unit increases or the presence vs. absence of the characteristic

## Discussion

In a population of UK community-dwelling older adults, we found that as expected being in a higher SPR category was associated with increased odds of reporting at least one fracture since the age of 45 years, when adjusting for age and sex only. This would appear appropriate as individuals who have already sustained a fracture are at the highest risk of suffering another [43], and this observation suggests that patients are aware of this. Given that previous studies found that being alone and socially isolated, having poor self-efficacy, and being depressed are associated with an increased risk of fracture [12–17] as well as poor self-rated health [7–9], we wanted to see whether these same factors may also play a role in SPR. However, further individual adjustment for social isolation, GSE score, and self-reported anxiety/depression did not affect the association between SPR and previous fracture, although this association was attenuated after adjustment for lifestyle and medical history.

Our findings suggest that, in our population sample, SPR was independent of social and psychosocial factors. Based on previous literature, we had hypothesized that being isolated, not being confident about one’s own ability to cope with everyday challenges and tasks of life, or feeling anxious/depressed might be associated with increased concerns about one’s own health risks, including the risk of fracture. We did not find evidence to support our hypothesis. While it is possible that no association exists, we should be mindful that this could be due to the low prevalence of some of the psychosocial risk factors considered in our study and the modest sample size used for analysis: previous literature suggests that 50% of the worldwide population aged over 60 is at risk of becoming socially isolated [44], while in the current study this prevalence was below 18%. By contrast, anxiety/depression was self-reported by 24.1% of our population sample, similar to the 22% prevalence previously found in a larger subset of the HCS [45]. Studies in larger samples where social isolation or loneliness is more common may still be appropriate.

While adjustment for social and psychosocial factors did not affect the relationship between SPR and previous fracture, interestingly we found that adjustment for risk factors not included in FRAX, such as prudent diet score, attenuated the association. We also found that higher quality of diet was associated with decreased odds of being in a higher SPR category. To our knowledge, no previous study has reported an association between diet quality and SPR. While adequate nutrition, and specifically adequate intake of calcium, protein and other vitamins and minerals, is known to be very important for maintaining musculoskeletal health [18–22], it is not apparent why following a higher quality diet would necessarily be related to lower odds of having a higher SPR. However, it is possible that a wider understanding of the potential benefit of adequate nutrition for musculoskeletal health accompanied by an adequate intake of nutrients known to be important for bone health can lead some participants to feel at a lower risk of fracture compared to others. It is also possible that participants with better diet quality were generally healthier, more active, and not taking medications that might influence their SPR. However, the association between diet quality and SPR was robust after adjustment for age, sex, BMI, smoking status, alcohol consumption, physical activity, number of comorbidities and current use of bisphosphonates, suggesting that these factors do not fully explain this association. These data warrant further study, possibly through qualitative work, to understand the relationships between dietary choice and SPR specifically. We also found that individual adjustment for other factors not included in FRAX, namely comorbidity, and use of bisphosphonates attenuated the association between SPR and previous fracture and that these factors were also associated with SPR. These findings are consistent with previous work in the Global Longitudinal Study of Osteoporosis in Women [6].

Our study has some limitations. First, our population sample may not be entirely representative of the wider UK population, as all participants were born in the county of Hertfordshire, were still living in their homes, and were all Caucasian. However, it has been previously demonstrated that the HCS is representative of the general population in terms of anthropometric body build and lifestyle factors (e.g., smoking and alcohol intake) [46], although a ‘healthy’ responder bias is evident within the HCS [29]. Second, the cross-sectional design of our analyses does not permit interpretations about the direction of causality. Third, the fairly small sample size prevented a robust stratified analysis of relationships between SPR and previous fracture according to levels of other characteristics, such as psychosocial factors. Fourth, a high number of variables were self-reported in our study and, therefore, recall bias cannot be ruled out. Finally, a further limitation of this study is the fact that fractures could not be captured for part of the time window between 2017 and 2019–2020 as only fractures over the previous 12 months were asked at the latter time point. On the other hand, our study has a number of strengths: while previous studies on SPR have focused exclusively on postmenopausal women [2, 6, 47–50], our population sample consisted of both men and women. In addition, we used validated tools where appropriate. For example, the LSNS-6 is a reliable tool for the assessment of social isolation [51].

## Conclusion

Consisting of a single simple question, the use of SPR alongside objective measures such as FRAX might be beneficial when assessing fracture risk in older adults as it might account for factors which although not included in FRAX, can be relevant to actual fracture risk. While adjustment for social and psychosocial factors did not affect the relationship between SPR and previous fracture, we found that individual adjustment for factors not currently included in FRAX, namely diet quality and number of comorbidities, attenuated this relationship. Our findings with regard to diet quality were unexpected and as such warrant further consideration as they suggest that dietary choice may be strongly linked to an individual’s perception of risk for osteoporosis, and possibly also other conditions.

## Data Availability

Hertfordshire Cohort Study data are accessible via collaboration. Initial enquires should be made to EMD (Principal Investigator). Potential collaborators will be sent a collaborators’ pack and asked to submit a detailed study proposal to the HCS Steering Group.
